# Synthesis of odorants in flow and their applications in perfumery

**DOI:** 10.3762/bjoc.18.76

**Published:** 2022-06-27

**Authors:** Merlin Kleoff, Paul Kiler, Philipp Heretsch

**Affiliations:** 1 Freie Universität Berlin, Institut für Chemie und Biochemie, Fabeckstr. 34–36, 14195 Berlin, Germany; 2 PK Perfumes, Menifee, California, United States of America; 3 Leibniz Universität Hannover, Institut für Organische Chemie, Schneiderberg 1B, 30167 Hannover, Germany

**Keywords:** flow chemistry, fragrances, odorants, scents, terpenes

## Abstract

Continuous flow technology is a key technology for sustainable manufacturing with numerous applications for the synthesis of fine chemicals. In recent years, the preparation of odorants utilizing the advantages of flow reactors received growing attention. In this review, we give an overview of selected methods for the synthesis of odorants in flow, including heterogeneously catalyzed reactions, gas reactions, and photochemical C–H functionalization processes. After a brief introduction on types of odorants, the presented odorant syntheses are ordered according to the main odor families “fruity”, “green”, “marine”, “floral”, “spicy”, “woody”, “ambery”, and “musky” and their use and importance for perfumery is briefly discussed.

## Introduction

The history of odorants goes back to ancient cultures such as the Egyptian around 5000 BC where resins of incense, opoponax, and myrrh were burnt for religious purposes [1]. Today, “the art of perfumery is closely connected to [synthetic] chemistry“, as outlined by Jean-Claude Ellena, the former master perfumer of Hermès [2]. In fact, the development of perfumes and cosmetics is strongly driven by the development of new odorants with unprecedented scents or superior physical properties [3–8]. Ernest Beaux, creator of Chanel *No. 5*, even claimed that “the future of perfumery is in the hand of chemistry” [3]. In addition, the industrial synthesis of odorants is the only way to provide them in sufficient quantities when natural sources are rare, or their production is unethical as it is the case for ingredients obtained from animals such as musk or civet [9–10].

In recent years, flow chemistry has enriched organic synthesis as an enabling technology to realize reactions that are impossible in batch or to provide products in higher purity avoiding expensive purification procedures [11–18]. Given the superior heat-, mass-, and phototransfer in microreactors, flow chemistry has been outlined as a central tool for sustainable manufacturing [18]. Utilizing the virtue of flow chemistry, more and more methods for the preparation of odorants in flow are developed. Recently, Baxendale and co-workers reviewed techniques and apparatus tailored to the synthesis of flavors and fragrances [19]. In this review, we want to give an overview of selected flow protocols for the synthesis of various odorants and highlight their role for perfumery. All quotations of percentages of these raw materials in perfumes are in the concentrate formula, before dilution, and are taken from GC/MS analyses.

## Review

### Classification of odorants

**Figure 1:**
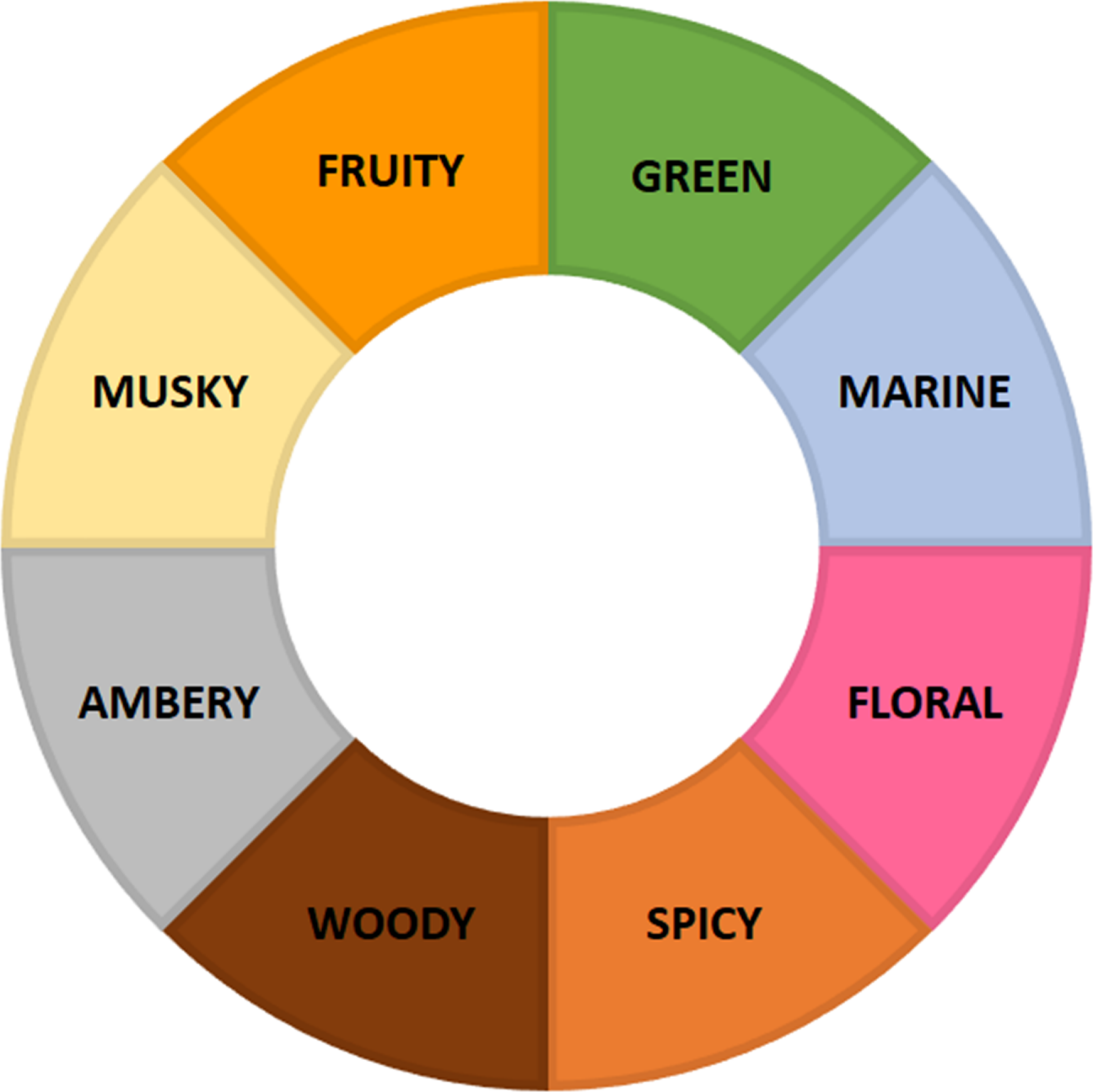
The olfactory spectrum wheel ordering different types of odorants from fruity to musky.

As there are many different types of scents, there are various classifications for fragrances. In this review, fragrances are ordered from “fruity” to “musky” following the olfactory spectrum wheel developed by Kraft and co-workers (Figure 1) [3–4]. It has to be noted that this is only a simple and subjective classification; most odorants belong to multiple categories.

**Figure 2:**
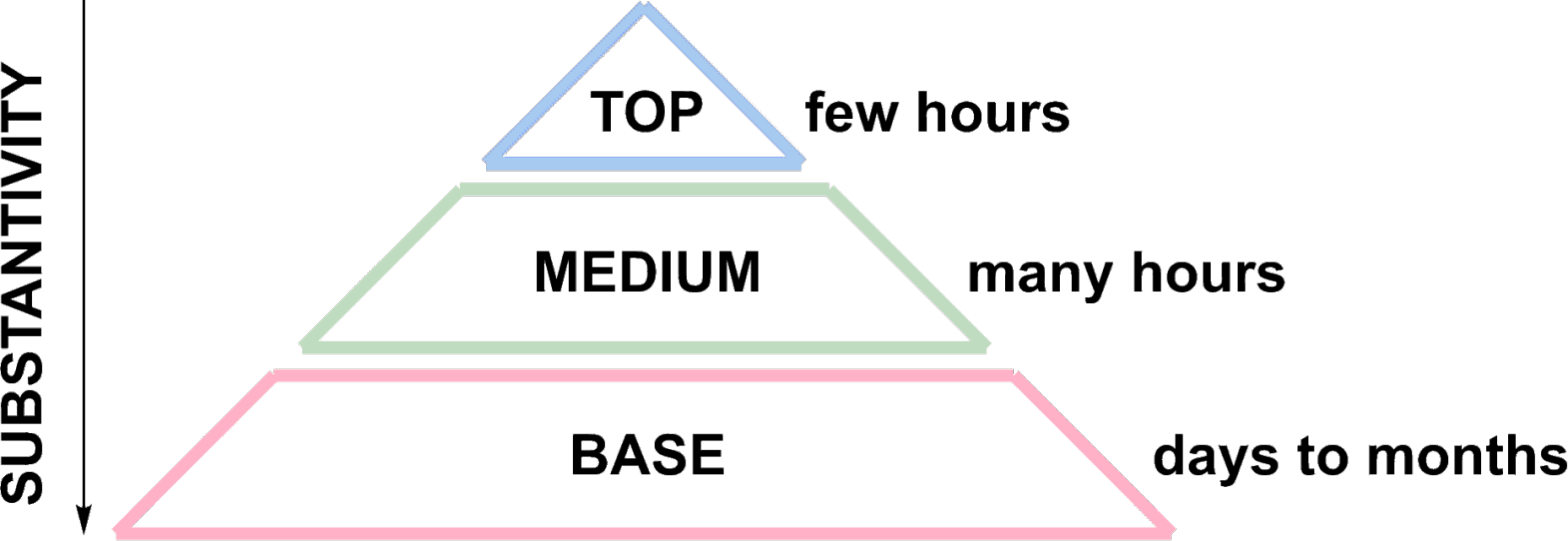
Classification of odorants as “top note”, “middle note” and “base note” depending on their substantivity.

Depending on the vapor pressure and consequently the perceptibility of an odorant on a paper strip (the so called “substantivity”), it can be ordered in a pyramid (Figure 2) as a “top note” (substantivity of up to a few hours), a “middle note” (substantivity of many hours), or a “base note” (substantivity of days up to weeks) [2,20]. Typically, fresh and citric odorants, e.g., limonene, are top notes, while warm and sweet odorants such as vanillin are base notes [21]. However, these classical categories have been partially overcome by synthetic odorants. For example, hedione (methyl dihydrojasmonate), one of the most important odorants of modern perfumery, has a fresh, citric and slightly floral scent – but is commonly categorized as a middle note with a substantivity of 72 h [22].

Most professional perfumes are well-balanced mixtures of top, middle, and base notes. While top notes have a “diffusive” effect on a perfume increasing its volatility, base notes may serve as “fixatives” reducing the volatility of a perfume and, thus, increasing its longevity. Notably, there are odorants (in particular amber notes) which are base notes serving as fixatives but also enhancing the perceptibility of a perfume [23].

### Fruity odorants

**Scheme 1:**
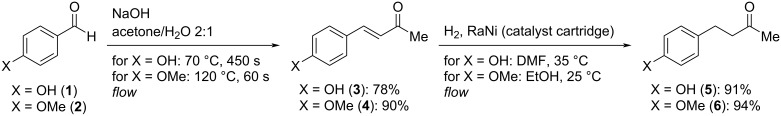
Synthesis of raspberry ketone (**5**) and raspberry ketone methyl ether (**6**) in two steps in flow.

One of the most important odorants giving raspberries their characteristic scent is the so-called “raspberry ketone” (**5**) having a “sweet, fruity, and warm odor” which is frequently used for fruity perfumes and as a flavor [9]. It is prominently used in, e.g., Tom Ford: *Tuscan Leather* along with notes of leather, muguet, and thyme, defining the character of this scent. The related methyl ether **6** (“raspberry ketone methyl ether”) is also used as odorant but is, in contrast to raspberry ketone (**5**), “intensely sweet, floral” and only “slightly fruity” [9]. Kappe and co-workers disclosed an access to both odorants in a two-step synthesis (Scheme 1) [24]. In the first step, 4-aryl-3-buten-2-ones **3** and **4** are prepared via aldol condensation of the corresponding aldehydes **1** and **2** and acetone in 78–90% yield with a productivity of up to 0.35 kg/h for enone **4**.

In the second step, the obtained 4-aryl-3-buten-2-ones **3** and **4** are selectively hydrogenated in flow using a packed-bed reactor with Raney nickel as catalyst affording raspberry ketone (**5**) in 91% yield and raspberry ketone methyl ether (**6**) in 94% yield, respectively. For compound **6**, both individual steps were combined for a two-step aldol condensation/hydrogenation flow sequence providing raspberry ketone methyl ether (**6**) on a gram scale in 75% overall yield. Interestingly, also alternative flow protocols for the synthesis of 4-aryl-3-buten-2-ones **3** and **4** were first developed on small scale under microwave batch conditions to reach short reaction times of 1–10 min and subsequently translated to scalable flow processes [24].

**Scheme 2:**
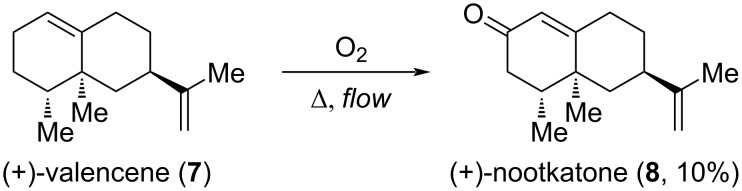
Autoxidation of (+)-valencene (**7**) to (+)-nootkatone (**8**) under catalyst and solvent-free conditions in a segmented flow.

While raspberries have a fruity and “berry” scent which is typically associated with the color red, the scent of citrus fruits is placed more between the fruity and the green notes. Among the odorants found in citrus fruits, such as oranges and grapefruits, (+)-nootkatone (**8**) is one of the most powerful odorants having a “sweet, citrusy” scent and a good substantivity [9]. However, (+)-nootkatone (**8**) is relatively expensive as it has to be extracted from grapefruits or prepared by, e.g., oxidation of (+)-valencene (**7**) (using toxic di-*tert*-butyl chromates), which is isolated from the essential oil of oranges [9]. In 2014, Neuenschwander and Jensen reported a flow setup for the catalyst and solvent-free oxidation of (+)-valencene (**7**) with molecular oxygen at elevated temperatures providing (+)-nootkatone (**8**) in 10% yield (Scheme 2). In this setup, neat (+)-valencene (**7**) is mixed with a stream of oxygen resulting in the formation of a segmented gas–liquid flow. In segmented flow a higher surface-to-volume ratio is achieved and toroidal currents occur within the liquid slugs which result in a continuous mixing of the liquid slugs. Therefore, the reaction proceeds up to 100 times faster in flow than under conventional batch conditions [25].

**Scheme 3:**
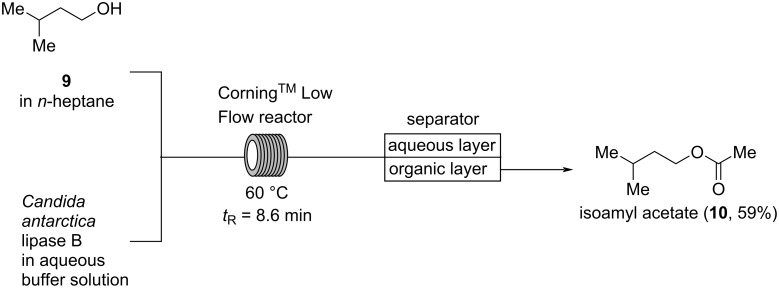
Enzyme-catalyzed acetylation of isoamyl alcohol (**9**) in a biphasic *n*-heptane/water mixture utilizing a Corning^TM^ Low Flow reactor.

Although, pure isoamyl acetate (**10**) has a “pronounced, fruity-fresh odor” which is “slightly nauseating”, it is “in dilution reminiscent of pear, banana, [and] apple” making it useful for perfumery in small doses [9]. For instance, it is appearing in both vintage Geoffrey Beene: *Grey Flannel* and Georgio Armani: *Acqua di Gioia eau fraiche* at 0.04%. Žnidaršič-Plazl and co-workers developed a method for the acetylation of isoamyl alcohol (**9**) catalyzed by *Candida antarctica* lipase B (Scheme 3) [26]. A biphasic system consisting of *n*-heptane and an aqueous buffer solution is used and efficiently mixed in a Corning AFR^TM^ Low Flow reactor providing a fine dispersion of the reaction mixture and, thus, a large interface between the phases. Subsequently, the biphasic system is directly separated, employing a PTFE membrane separator, to afford a solution of isoamyl acetate in *n*-heptane, while the aqueous layer containing the lipase could be recycled. At 60 °C with a residence time of 8.6 min isoamyl acetate (**10**) is obtained in 59% yield according to GC analysis [26]. Related methods for the enzyme-catalyzed acetylation of isoamyl alcohol (**9**) have been developed utilizing biphasic systems, supercritical carbon dioxide as a solvent, or packed-bed reactors with immobilized enzymes [27–31].

**Scheme 4:**
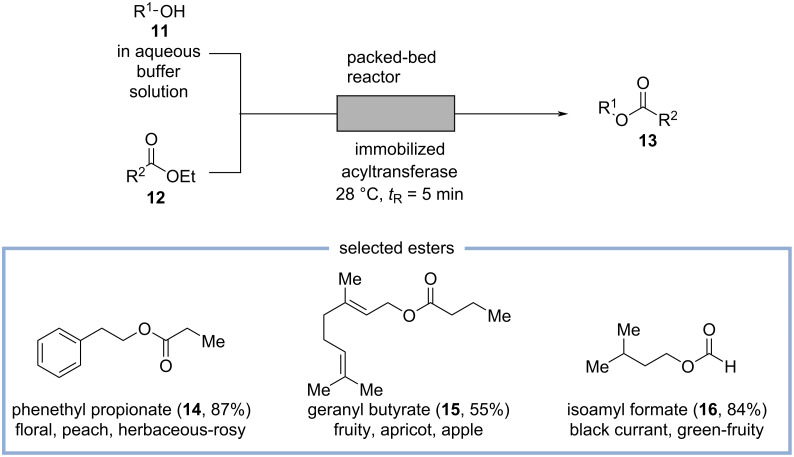
Esterification of alcohols by transesterification, catalyzed by immobilized acyltransferase in a packed-bed reactor and the corresponding odor profiles of selected examples [9,32].

More recently, Paradisi and co-workers disclosed a more general access to a variety of esters with mainly fruity and floral odorants by transesterification of acyl donors of structure **12** to the corresponding alcohols **11** using an immobilized transferase obtained from *Mycobacterium smegmatis* (Scheme 4) [32]. A solution of the acyl donor **12** in ethyl acetate and an aqueous buffer solution of the corresponding alcohols **11** are mixed in a T-piece and the resulting segmented flow is pumped through a packed-bed reactor containing the immobilized transferase. The reaction mixture is directly analyzed by GC, or, as demonstrated for the preparation of phenylethyl acetate, further diluted with ethyl acetate, and the biphasic system is separated in flow providing phenylethyl acetate in 82% isolated yield. Using this method, a variety of 2-phenylethyl-, cinnamyl-, geranyl-, *n*-hexyl-, and isoamyl esters with mainly fruity odor profiles are obtained in moderate to excellent yields. Some selected esters (**14**–**16**) and their odor profiles are shown in Scheme 4 [32].

Related methods for the esterification of natural occurring alcohols, such as geraniol, utilizing immobilized enzyme-catalysis in packed-bed reactors were developed by the groups of de Souza and Yadav [33–34].

**Scheme 5:**
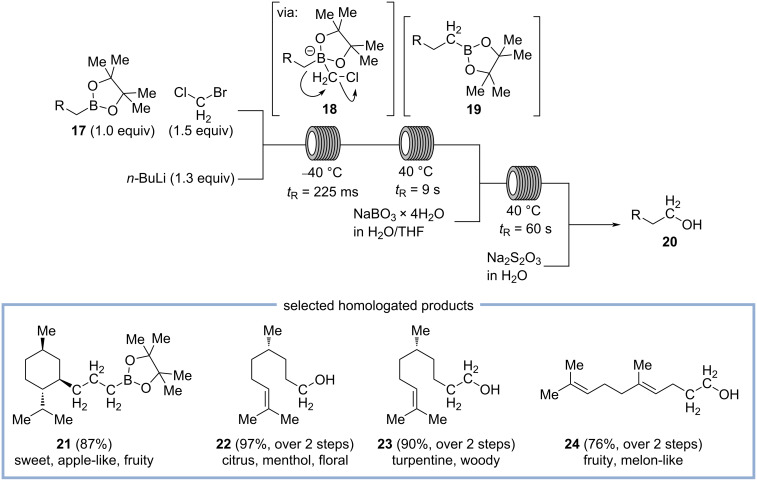
Synthesis of homologated alcohols **20** by iterative homologation of terpenyl boronate esters **17** followed by oxidation in flow.

Very recently, Kirschning and co-workers presented a general method for the Matteson reaction in flow, allowing iterative homologation of various terpene boronate esters **17**, which are subsequently oxidized to the corresponding alcohols **20** (Scheme 5) [35]. In the first step, a solution of terpenyl pinacol boronates **17** and bromochloromethane in tetrahydrofuran is mixed with *n*-butyllithium in *n*-hexane at −40 °C. By using a specifically designed, 3D-printed micromixer made from stainless steel, ultrafast mixing of both solutions is achieved within milliseconds initializing bromine–lithium exchange of bromochloromethane to generate (chloromethyl)lithium. This carbenoid species readily reacts with terpenyl pinacol boronates **17**, resulting in the formation of intermediate **18**, which undergoes 1,2-anionotropic rearrangement to the homologated pinacol boronate **19**. As the rearrangement is a much slower process, the reaction mixture is passed through a second reactor at elevated temperature with a residence time of 9 s to allow full conversion to the homologated pinacol boronate **19**. This species can then be directly pumped to a second homologation reactor module (and then, if desired, even to a third) to reiterate homologation. The resulting reaction mixture is either collected directly to provide the homologated pinacol boronates **19**, e.g., menthol-derived compound **21**, which was found to have a “rather sweet, slightly apple-like, fruity odor”. Alternatively, the reaction mixture is pumped to an oxidation module in which the pinacol boronates are mixed with a solution of sodium perborate in water/tetrahydrofuran to perform oxidation to the corresponding alcohols **20** at 40 °C in 60 s. In order to quench remaining oxidants, the reaction mixture is combined with an aqueous solution of sodium thiosulfate before it is collected. Among the prepared alcohols, a few compounds were found to have interesting olfactory properties. Alcohol **22** has a citrus and menthol note, while its homolog **23** shows a turpentine-like, woody scent. In contrast, alcohol **24**, which is a homolog of geraniol, has a strong fruity, melon-like odor profile [35].

### Green odorants

To the family of green odorants belong those having a scent that is reminiscent of leaves and grasses, but also odorants that are minty, camphorous, or resinous. Peppermint is a fresh, cold, and quite clean top note that is mostly employed in men’s fragrances, both in classical (Davidoff: *Cool Water* at approx. 0.2%) and modern fragrances (Chanel: *Allure Homme Sport*, Jean-Paul Gaultier: *Le Mâle* at approx. 0.01%). However, small doses of mint notes give a desirable sparkle even to female fragrances, e.g., Parfums de Marly: *Delina Exclusif*, and for topnote blends for tuberose flower accords used in fragrances. The most important mint notes are certainly menthone and menthol, but occasionally (*S*)-α-phellandrene gives better results in a perfume [9].

**Scheme 6:**
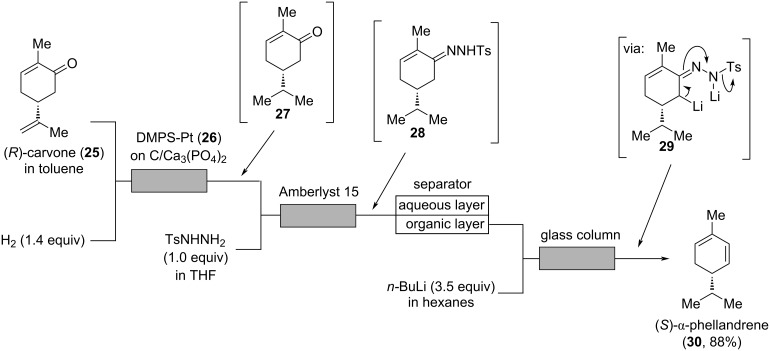
Sequential three-step synthesis of (*S*)-α-phellandrene (**30**) from (*R*)-carvone (**25**) via selective hydrogenation to enone **27**, condensation to hydrazone **28** and subsequent Shapiro reaction. DMPS = dimethylpolysilane-modified platinum catalyst; Ts = tosyl.

“When absolutely pure”, (*S*)-α-phellandrene “has a pleasant, fresh-citrusy, and peppery-woody odor with a discretely mint note” [9]. Very recently, Kobayashi, Ishitani, and co-workers described a three-step sequential continuous flow process for the synthesis of (*S*)-α-phellandrene (**30**) from (*R*)-carvone (**25**, Scheme 6) [36].

In the first step, a solution of (*R*)-carvone (**25**) in toluene is merged with a stream of hydrogen and the resulting segmented flow is passed through a column reactor containing a dimethylpolysilane-modified platinum catalyst (DMPS-Pt, **26**), immobilized on carbon/calcium phosphate. At a temperature of 25 °C using 1.4 equivalents of hydrogen with a pressure of 1 bar, a good selectivity for the hydrogenation of the external alkene is achieved providing enone **27**. The reaction mixture containing enone **27** is then mixed with tosylhydrazone and passed through a column with sulfonic acidic resin Amberlyst-15 to catalyze the formation of hydrazone **28**. As one equivalent of water is formed in this condensation process, which is detrimental for the subsequent Shapiro reaction, water is continuously removed by in-line separation of the reaction mixture using a PTFE-membrane separator. The organic layer is then mixed with a solution of *n*-butyllithium in hexanes to initiate the Shapiro reaction of hydrazone **28** proceeding supposedly via dilithiated intermediate **29**. As the nitrogen produced in the reaction increases the volume of the reaction mixture and therefore is drastically shortening the residence time, a cooled glass column with the flow direction oriented against gravity is utilized. In this way, nitrogen bubbles can move to the top of the column, while the liquid reaction mixture remains below. At the outlet of the column, the reaction mixture is directly collected in a stirred flask containing water to quench the reaction. After filtration through activated aluminum, (*S*)-α-phellandrene (**30**) is obtained in a high yield of 88% over three steps on a 30 g scale corresponding to a productivity of 0.887 mol/day [36].

**Scheme 7:**
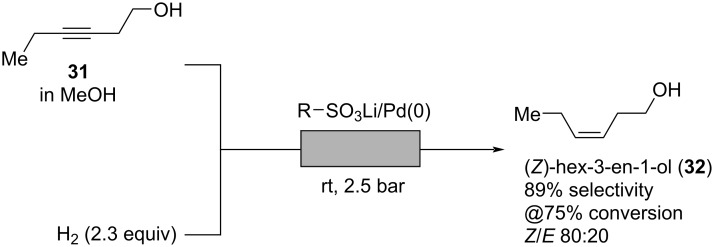
Selective hydrogenation of alkyne **31** to “leaf alcohol” **32** employing a solid-supported palladium catalyst.

The smell of leaves and freshly cut green grass can mainly be traced back to (*Z*)-hex-3-en-1-ol (**32**) (so-called “leaf alcohol”), an important odorant with an intense green and grassy odor “often used along with geranium oil, galbanum, oakmoss, lavender, and mint oils” [4,9]. The freshness that comes from green notes such as (*Z*)-hex-3-en-1-ol, 2,4-dimethylcyclohex-3-ene-1-carbaldehyde, and (*E*,*Z*)-2,6-nonadien-1-al are nearly ubiquitous in modern perfumery for both women and men, even appearing in dark or woody fragrances such as Hugo Boss: *Soul*. In 2012, Barbaro and co-workers developed a synthesis for alkene **32** by selective hydrogenation of the corresponding alkyne (Scheme 7) [37]. Instead of using a Lindlar catalyst containing toxic lead salts, selectivity is achieved by the improved reaction control in flow. A solution of alkyne **31** in methanol is mixed with a stream of hydrogen and pumped at room temperature and 2.5 bar through a tubular glass column containing a Dowex-supported palladium catalyst. Optimization of process parameters revealed that at a conversion of 75% a good selectivity of 89% for hydrogenation of alkyne **31** to alkene **32** is achieved affording a mixture of (*Z*)- and (*E*)-isomers in a ratio of 80:20 [37].

### Floral odorants

Floral notes, such as rose, jasmine, orange blossom, or lavender, are typically middle notes defining the “heart” of many perfumes. To create a jasmine note, the synthetic odorant jasmonal (**35**) can be used which has an “oily-herbaceous and somewhat floral odor, reminiscent of many types of natural flowers, but mostly of jasmine, gardenia, and tuberose.” It is “used very extensively in perfumes” and “soap perfumes” to introduce “jasmine-like floralcy when accompanied by more volatile chemicals of floral character”, while assisting “in fixation of the fragrance” due to its relatively high boiling point of 285 °C. It is industrially produced by an aldol condensation of heptanal (**34**, obtained from castor oil) and benzaldehyde (**33**). In the industrial process, stoichiometric amounts of sodium- or potassium hydroxide are used resulting in the formation of large quantities of undesired side products, e.g., enone **36**, the aldol condensation product of two molecules of heptanal [9,38].

**Scheme 8:**
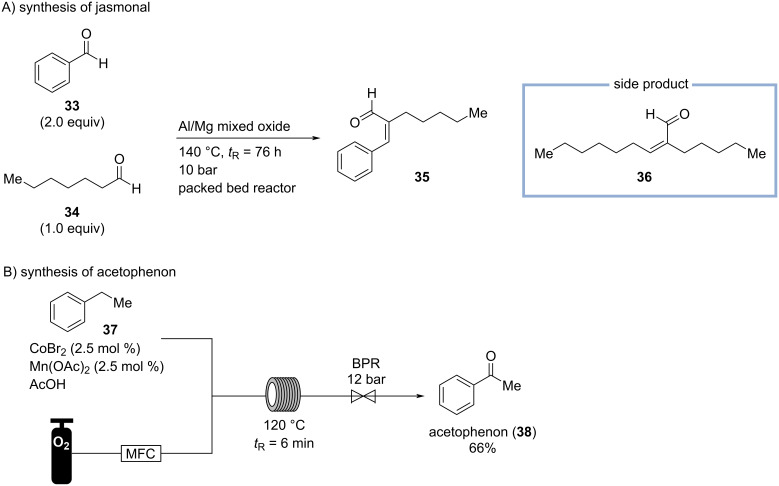
A) Synthesis of jasmonal (**35**) by crossed aldol condensation of benzaldehyde (**33**) and heptanal (**34**) in a packed bed reactor containing a magnesium/aluminum mixed oxide catalyst. B) C–H oxidation of ethylbenzene (**37**) to acetophenone (**38**) enabled by a MC-system as catalyst and molecular oxygen. BPR = back pressure regulator; MFC = mass flow controller.

Therefore, Gholami and co-workers developed a flow protocol for the synthesis of jasmonal (**35**) by aldol condensation of heptanal (**34**) and benzaldehyde (**33**) utilizing a magnesium-aluminum mixed oxide catalyst in a fixed bed reactor (Scheme 8A) [38]. To suppress the formation of side product **36**, an excess of benzaldehyde is used. At 140 °C and with a long residence time of 76 h, a moderate heptanal (**34**) conversion of 36% was achieved providing jasmonal (**35**) in a selectivity of 41% [38].

One of the least expensive floral odorants is acetophenone (**38**), having a “pungent-sweet odor, in dilution resembling that of hawthorn or a harsh orange-blossom type“. Acetophenone appears in vintage Geoffrey Beene: *Grey Flannel* at 0.14%, and Shiseido: *Zen* and Gap: *Om* at approx. 0.014%. In 2013, Roberge, Kappe, and co-workers investigated the C–H oxidation of ethylbenzene (**37**) to acetophenone with oxygen as an oxidant (Scheme 8B) [39]. The process is performed at 120 °C at 10 bar with a residence time of 6 min, and catalyzed homogenously utilizing the established “MC-system” (manganese/cobalt/bromide) in a heated tube reactor. Remarkably, acetophenone is obtained in a good yield of 66% and in 96% purity without purification, while other oxidation products are formed in only small quantities further exemplifying the selectivity of the flow process [39].

### Spicy odorants

**Scheme 9:**
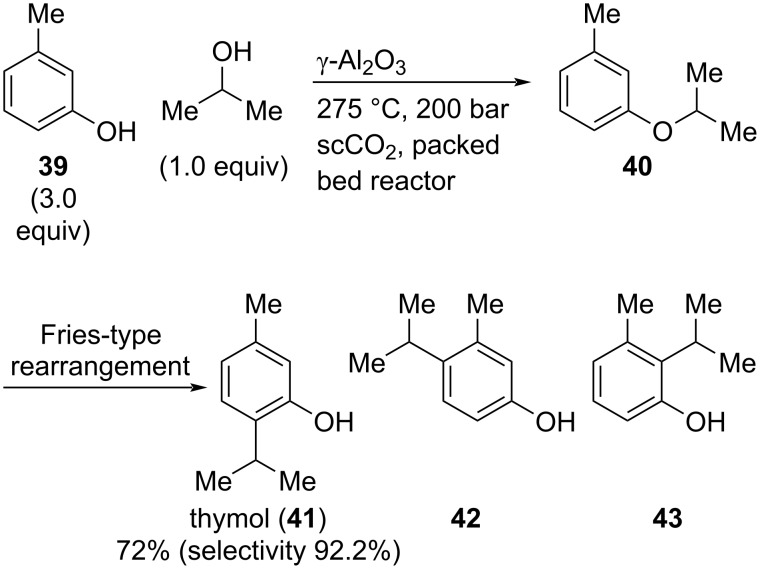
Synthesis of thymol (**41**) from *m*-cresol (**39**) and isopropyl alcohol via Fries-type rearrangement of ether **40**.

Similar to green notes in breadth and variety of odor profiles, are many spice oils and related molecules used in fragrances. Many of these molecules found in natural spice oils are terpenes, which belong to the top notes. Some molecules get to the middle notes, and, very rarely, spice materials reach the base notes. Thyme is occasionally used in soapy perfumes and detergents “where its power and freshness can introduce a hint of medicinal notes” [10]. This material is employed in, e.g., Tom Ford: *Tuscan Leather* to introduce a slightly medicinal and spicy note complementing the leathery and smoky notes (so called “white” thyme appears at approx. 0.25% in *Tuscan Leather*). One of the main ingredients of thyme is thymol (**41**) which has a sweet-medicinal and warm odor; interestingly, it is also strongly antiseptic [9]. In 2005, Poliakoff and co-workers developed a synthesis of thymol by alkylation of *m*-cresol (**39**) in supercritical carbon dioxide (scCO_2_) using γ-Al_2_O_3_ in a packed-bed reactor (Scheme 9) [40].

In the presence of Brønsted-acidic Nafion SAC-13, alkylation of *m*-cresol with isopropanol proceeds via a Friedel–Crafts-type mechanism in much lower selectivity. In contrast, the authors proposed that employing γ-Al_2_O_3_ as Lewis acid catalyst, reaction of **39** and isopropanol leads to isopropyl ether **40**. This intermediate undergoes a Fries-type rearrangement resulting in the formation of thymol (**41**) along with its regioisomers **42** and **43**. However, using an excess of isopropanol and a relatively low concentration of the organic substrates in scCO_2_ (5% w/w), thymol (**41**) is produced in a good yield (72%) and selectivity (92.2%) as shown by GC. Interestingly, it was found that a higher substrate concentration is disadvantageous, as water is formed in the process which decreases the activity of the catalyst. Thus, at a higher substrate concentration, the water removal by scCO_2_ is not sufficient, thereby lowering both, yield and selectivity, of the process [40].

The first synthesis of coumarin by Perkin in 1868 was a breakthrough in the history of natural odorant synthesis [2]. Coumarin (**46**) has a sweet, slightly spicy, and hay-like scent [9,41]. It was extensively used in Houbigant: *Fougère Royal* (1882, appearing at approx. 10%), a perfume which has lent its name to a whole family of perfumes (*fougère perfumes*) [2]. Despite the molecular size of coumarin, it is often considered as a base note, but higher dosages can bring it into the middle notes.

**Scheme 10:**
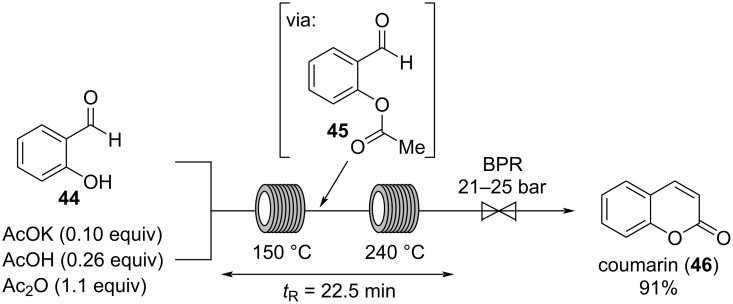
Preparation of coumarin (**46**) by reaction of salicylaldehyde (**44**) with potassium acetate, acetic acid, and acetic anhydride at elevated temperature in two separated tube reactors.

In 2015, Guo and co-workers reported a flow procedure for the synthesis of coumarin (**46**) following the Perkin synthesis (Scheme 10) [42]. Salicylaldehyde (**44**) is mixed with a solution of potassium acetate and acetic acid in acetic anhydride. The reaction mixture is pumped through two separated tube reactors at 150 °C and 240 °C, respectively, proceeding with a combined residence time of 22.5 min. The authors propose that the reaction does not follow the mechanism of the Perkin process but proceeds via acylation of salicylaldehyde (**44**) to intermediate **45**, which forms coumarin (**46**) in an intramolecular aldol cyclization. Therefore, *O*-acylation of salicylaldehyde (**44**) is completed at 150 °C before the aldol condensation is initiated at 240 °C. While at lower temperatures, the aldol condensation proceeded incompletely, increasing the temperature to 250 °C lead to clogging of the reactor probably due to formation of phenolic resins as byproducts. In contrast, when the reaction is performed at 240 °C in one tube reactor, the reaction gives incomplete conversion and the yield of coumarin (**46**) drops to 21%. The authors proposed that under these conditions the reaction proceeds via the Perkin process, which is significantly slower than the *O*-acylation/aldol sequence [42].

**Scheme 11:**
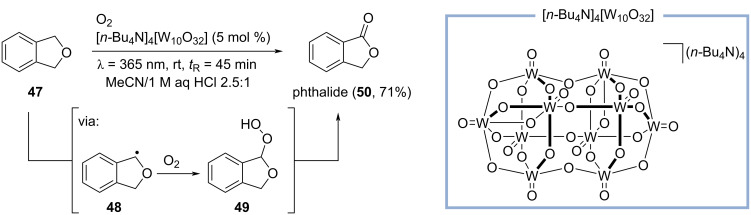
Synthesis of phthalide (**50**) by photoinduced decatungstate catalysis.

An odorant that is somewhat similar to coumarin is phthalide (**50**), having a sweet and powdery scent reminiscent of coconut and tonka bean [43]. Phthalide can be considered as a top note type of coumarin. Recently, Noël and co-workers developed a method for the photochemical, decatungstate-catalyzed C–H oxidation of activated and unactivated alkanes, including the transformation of isodihydrobenzofuran (**47**) to phthalide (**50**, Scheme 11) [44]. In this reaction, the decatungstate anion is activated by irradiation in a 3D-printed tube reactor using LED light with a wavelength of λ = 365 nm. It is assumed, that the photoexcited state of the decatungstate anion generates carbon-centered radical **48** which is trapped in a segmented flow with molecular oxygen provided by a mass flow controller. Peroxide **49** is formed as intermediate which further reacts to phthalide (**50**) in 71% yield. This method efficiently utilizes the advantages of flow chemistry for photoreactions and reactions with gases providing shorter reaction times and improved scalability [44].

### Woody odorants

**Scheme 12:**
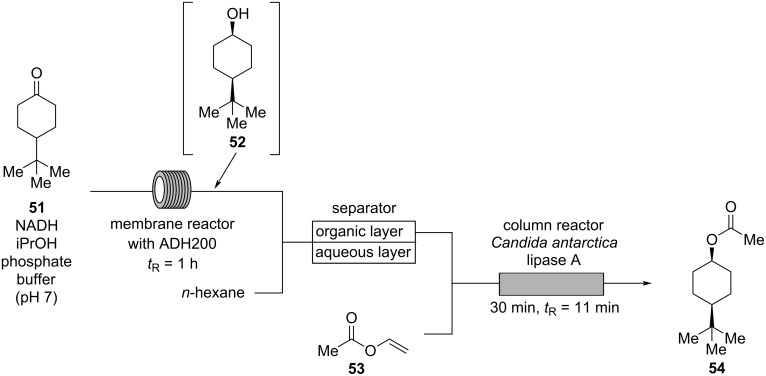
Synthesis of woody acetate (**54**) by reduction of cyclohexanone **51** and subsequent acetylation; ADH200 = alcohol dehydrogenase.

Woody odorants are widely used, especially in masculine perfumes. Some woody essential oils like cedarwood are relatively inexpensive, however, sandalwood oil has been overharvested for decades, and is now extremely expensive. Hence, for both expense and variety of woody type notes, there is a great demand of synthetic woody odorants. Among them, woody acetate (Vertenex, **54**) is an inexpensive ester with a “sweet, almost creamy-woody odor”. Typically, woody acetate (**54**) is sold as a mixture of the *cis*- and the *trans*-isomer. Interestingly, the *cis*-isomer of **54** has a “pronounced fruity note over the woody sweetness”, while the *trans*-isomer is weaker having a “dry, leathery” scent [9]. The commonly used concentration of woody acetate (**54**) in a perfume is 3–10%. Thus, Brenna and co-workers developed a *cis*-selective synthesis of **54** via a biocatalytic process in flow (Scheme 12) [45]. In the first step, a mixture of cyclohexanone **51**, NADH, and isopropanol in an aqueous phosphate buffer (pH ≈ 7) is pumped through a continuously stirred membrane reactor at 30 °C with a residence time of 1 h containing alcohol dehydrogenase (ADH200). In this process, cyclohexanone **51** is selectively reduced to the corresponding *cis*-alcohol **52** and subsequently mixed with *n*-hexane providing a biphasic mixture which is continuously separated in a membrane separator. The organic layer is mixed with vinyl acetate (**53**) and pumped through a column reactor containing *Candida antarctica* lipase A. At 30 °C and with a residence time of 11 min, acetylation of *cis*-alcohol **52** is mediated. After distillation, *cis*-woody acetate **54** is obtained in 89% isolated yield (de > 99%) on a gram scale [45].

Additionally, Noël and co-workers prepared (+)-sclareolide (3a,6,6,9a-tetramethyl-1,4,5,5a,7,8,9,9b-octahydronaphtho[8,7-*d*]furan-2-one), a rarely used aromatic odorant reminiscent of cedar and tobacco, by C–H oxidation of (−)-ambroxan (1,5,5,9-tetramethyl-13-oxatricyclo[8.3.0.0(4,9)]tridecane) using the same method as described above for the synthesis of phthalide (**50**, see Scheme 11) [44]. However, this synthesis is more of academic value for the research on C–H functionalizations as, in fact, (−)-ambroxan is industrially prepared by reduction of (+)-sclareolide [4].

### Musky odorants

Originally, musk was obtained from the gland of the musk deer and used as a powerful base note. Due to the high price of musks of natural origin, the vast majority of them is produced by chemical synthesis. Today, a plethora of synthetic musks with a broad structural and olfactory diversity are available, and commonly, musk molecules comprise between 20–40% of a fragrance. Typically, musk odorants are sweet, waxy, and “animalic”, bringing warmth and erogenous mystery to a perfume. However, many modern musks can also be fresh (e.g., Galaxolide, 4,6,6,7,8,8-hexamethyl-1,3,4,6,7,8-hexahydrocyclopenta[*g*]isochromene), fruity (e.g., Helvetolide, 2-(1-(3,3-dimethylcyclohexyl)ethoxy)-2-methylpropyl propionate, has a distinct pear note), powdery (e.g., Tonalide, 1-(5,6,7,8-tetrahydro-3,5,5,6,8,8-hexamethyl-2-naphthalinyl)ethanone), or show even unexpected notes, such as a metallic character reminiscent of hot iron (Habanolide, (12*E*)-oxacyclohexadec-12-en-2-one) [3–4,9,46–47].

**Scheme 13:**
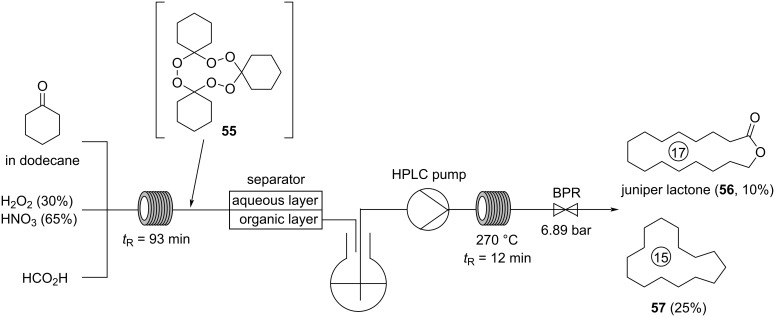
Synthesis of juniper lactone (**56**) by pyrolysis of triperoxide **55** generated by oxidation of cyclohexanone in flow.

Although, all musks of natural origin are macrocyles, most synthetic musks are polycyclic musks (PCM), while the fourth synthetic generation of musks are linear molecules [3–4]. In 1999, juniper lactone (**56**) was isolated from the flowers of orchids among with the structurally related and better known Ambrettolide [(*Z*)-7-hexadecen-16-olide], having a sweet odor with “great tenacity and fixative power” [9,48]. Notably, and already in 1970, Story and co-workers described that cyclic ketones can be reacted with hydrogen peroxide under acidic conditions to the corresponding triperoxides which form macrocyclic structures at high temperatures, e.g., juniper lactone (**56**) [49]. Despite the elegance of this access, the safety of this process is questionable complicating the large-scale production in batch. Very recently, Kirschning and co-workers developed a flow protocol to overcome these limitations and prepared various macrolactones from triperoxides by pyrolysis in an inductively heated flow reactor (Scheme 13) [50–51]. Due to the commercial relevance of juniper lactone (**56**), they designed a flow setup for a scalable and safe two-step synthesis of **56** from cyclohexanone.

In the first step, a solution of cyclohexanone in dodecane is mixed in a Q-piece with hydrogen peroxide, nitric acid, and formic acid and subsequently pumped at room temperature through a PTFE tube reactor with a residence time of 93 min. The resulting biphasic mixture is separated using a membrane reactor with a PTFE membrane. While the aqueous layer is directly quenched with an aqueous solution of sodium sulfite, the organic layer containing triperoxide **55** in dodecane is collected in a flask and directly pumped using an HPLC pump in an inductively heated tube reactor made from stainless steel 316L. In this reactor, the second step, i.e. pyrolysis of triperoxide **55** is achieved at 270 °C with a residence time of 12 min. The reaction mixture is collected and analyzed by GC/MS indicating formation of juniper lactone (**56**) in a yield of 10% along with cyclopentadecane (**57**, 25% yield) and other byproducts. Although, juniper lactone (**56**) is obtained in a relatively low yield, this protocol allows its scalable and straightforward synthesis from simple and inexpensive starting materials. The dangers associated with organic peroxides are significantly reduced by conducting both generation and pyrolysis of triperoxide **55** in flow reactors, while phase separation of the biphasic mixture containing triperoxide **55** is realized in a PTFE membrane reactor [50].

**Scheme 14:**
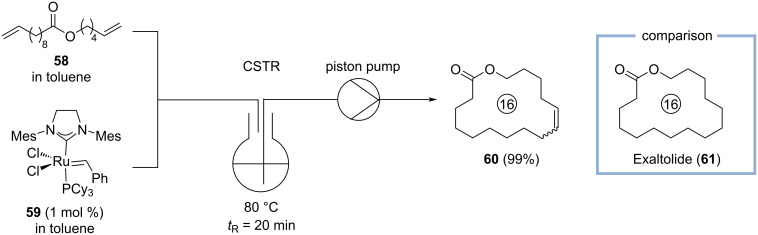
Synthesis of macrocyclic olefine **60** by ring-closing metathesis of diene **58** in a continuously stirred tank reactor (CSTR).

Since many macrocyclic musks (or their precursors) contain internal olefins, they are frequently prepared by ring-closing metathesis [3–4]. It is well described that metathesis reactions can be significantly accelerated in flow, as the boiling point of the solvent employed can be exceeded using back pressure regulators (BPRs) and formed gases (e.g., ethylene) can be easily removed employing tube-in-tube reactors [11]. Therefore, Roberge, Fogg, and co-workers investigated the advantages of continuously stirred tank reactors (CSTR) and tube reactors in comparison to the corresponding batch reaction for the ring-closing metathesis of diene **58**, producing macrocyclic olefin **60** (Scheme 14) [52]. Although, macrocycle **60** is not a commercial product, the corresponding saturated lactone **61** (Exaltolide), is “delicately animal, musky, and sweet” and regarded by many perfumers as one of the most elegant musks [9].

In batch and employing 1 mol % catalyst loading, the reaction reaches a maximum conversion of 82% at 80 °C after 10 min. When diene **58** and catalyst **59** are pre-mixed and pumped through a tube reactor, the ethylene released from the reaction results in a segmented flow. Therefore, the formed ethylene is not released from the tube reactor, hampering the reaction to reach full conversion as observed under batch conditions. In contrast, when a continuously stirred tank reactor was used, its head space was continuously flushed with argon to keep the concentration of ethylene in solution as low as possible. In this case, full conversion to macrocyclic olefin **60** (99% yield, detected be GC-FID) could be achieved after 20 min at 80 °C using 1 mol % of catalyst **59** [52].

**Scheme 15:**
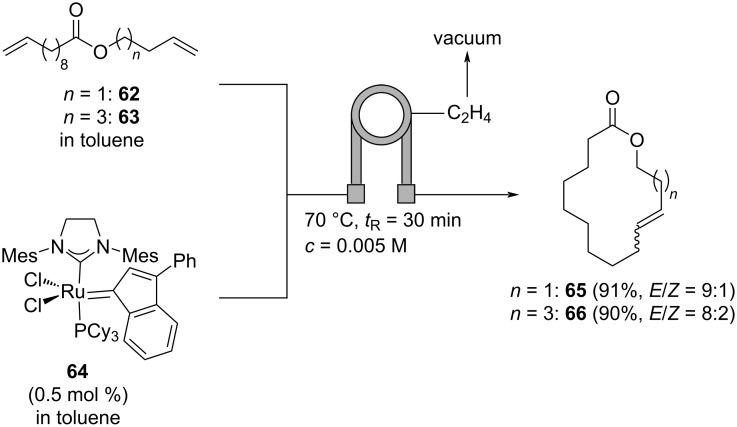
Synthesis of macrocycles **65** and **66** by ring-closing metathesis of dienes **62** or **63**, respectively, in a tube-in-tube reactor removing the formed ethylene.

Due to the fact that ethylene formed in the ring-closing metathesis can result in the formation of unstable ruthenium methylidene species, causing degeneration of the metathesis catalyst, the continuous removal of ethylene from the reaction mixture can be highly beneficial. Therefore, Skowerski and co-workers constructed a tube-in-tube reactor for the ring-closing metathesis of dienes **62** and **63** to macrocycles **65** or **66**, respectively, mediated by ruthenium catalyst **64** (Scheme 15) [53]. The substrate and the catalyst are mixed in a Q-piece and pumped through a tube reactor in which a smaller tube consisting of semipermeable Teflon AF2400 is placed. To the inner tube, vacuum is applied removing efficiently the ethylene that is formed in the reaction. Using a low concentration of the substrate to facilitate ring-closure and employing a moderate temperature of 70 °C and a residence time of 30 min, macrocycles **65** and **66** are obtained in excellent yields with a preference for the *E*-isomers as determined by GC/MS analysis. Although, macrocycles **65** and **66** are, to the best of our knowledge, not used as musks in perfumery, this work demonstrates the value of tube-in-tube reactors for the preparation of musk-like structures (in fact, **66** (*n* = 3) is an isomer of the widely used musk Habanolide, (12*E*)-oxacyclohexadec-12-en-2-one) [53].

**Scheme 16:**
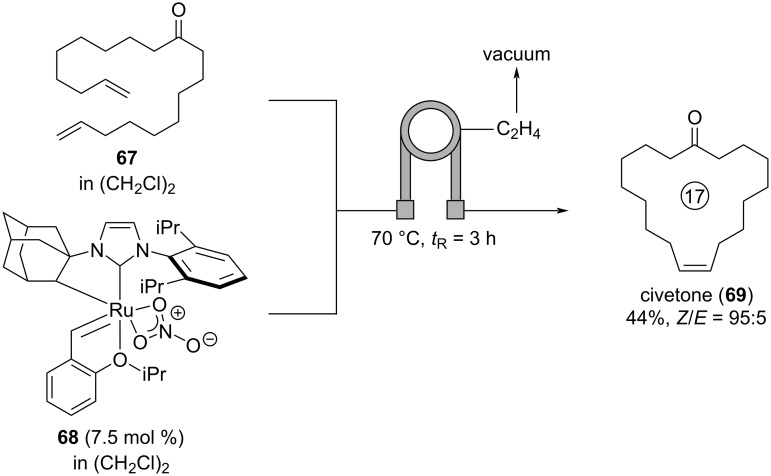
*Z*-Selective synthesis of civetone (**69**) enabled by metathesis catalyst **68** in a tube-in-tube reactor.

Civetone (**69**) is the key ingredient of natural civet, which is the glandular secretion of civet cats. Civet is an extremely long-lasting ingredient giving a fragrance a warm and animalic touch while enhancing floral notes [9]. Natural civet has been used in large quantities in classical perfumes such as Guerlain: *Jicky* (1889, used at approx. 0.6–1.0%) or Coty: *Chypre* (1917) [7]. Due to the high price of natural civet and as the civet cats suffer from captivity and the torturous (and sometimes even deadly) “extracting process“ of the civet oil, most perfumers deny using natural civet [10]. Therefore, synthetic replicates of civet have been developed which are constructed around civetone (**69**) as key ingredient. In most syntheses of civetone (**69**), the internal double bond is prepared in a ring closing metathesis providing a mixture of civetone (**69**) and the undesired *E*-isomer. More recently, Browne, Mauduit, and co-workers developed a *Z*-selective synthesis of civetone (**69**) in a tube-in-tube reactor (Scheme 16) [54]. Solutions of dialkene **67** and the *Z*-selective ruthenium catalyst **68** in 1,2-dichloroethane are mixed and pumped through a tube-in-tube reactor continuously removing the ethylene formed in the ring-closing metathesis. At 70 °C and with a residence time of 3 h, civetone (**69**) is formed in 44% isolated yield with a *Z*/*E* ratio of 95:5. Additionally, the authors developed an alternative synthesis of civetone (**69**) by metathesis of ethyl 9-decenoate and subsequent Dieckmann cyclization in flow, followed by a saponification and decarboxylation process in batch providing (*Z*)-civetone in 48% yield over three steps and with a *Z*-selectivity of >98% [54].

**Scheme 17:**
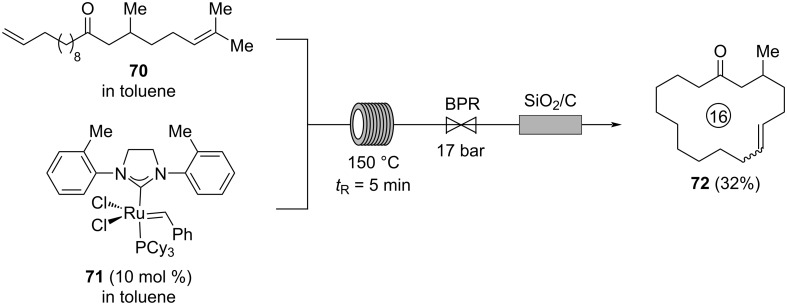
Synthesis of macrocyclic olefine **72** by ring-closing metathesis of diene **70**.

In contrast, Amorelli, Collins, and co-workers performed a ring-closing metathesis for the synthesis of macrocycle **72** from diene **70** at high temperatures of 150 °C in only 5 min without removal of formed ethylene (Scheme 17) [55]. Under these conditions, the employed Stewart–Grubbs catalyst **71** is completely decomposed but its decomposition products could efficiently be removed by passing the reaction mixture through a cartridge containing a mixture of silica and charcoal providing **72** in 32% yield at a productivity of 0.2 g/h. The macrocycle **72** was already synthesized by International Flavors & Fragrances (IFF) in 2013 and found to have a “strong musky odor” with “highly desirable properties in the top and middle notes that were described as feminine, smooth, creamy, warm, and comfortable” [55].

## Conclusion

Flow chemistry has evolved as a valuable tool for organic synthesis that simplifies upscaling and, in some cases, allows to overcome limitations in batch, while being safer and more sustainable. These advantages have been utilized for the preparation of various odorants, reaching from fruity and green odorants, which are typically small and volatile molecules, to macrocyclic musks with higher molar masses and boiling points.

In flow, photocatalyzed oxidations with molecular oxygen proceed in higher yields and with shorter reaction times, as it has been used for the synthesis of, e.g., phthalide (**50**). In contrast, when ethylene is formed in a ring-closing metathesis reaction for the preparation of macrocyclic musks, the efficient removal of ethylene by the means of a tube-in-tube reactor or a continuously stirred tank reactor (CSTR) can promote the reaction and suppress decomposition of the catalyst. In addition, reactions involving organometallics at low temperatures or instable intermediates, e.g., endoperoxide (**55**), benefit from the superior reaction control and safety profile of flow reactors. Many of the transformations in this review demonstrate the utilization of solid-supported reagents in cartridges that allow to avoid separation of the reagent after performing the respective reaction.

Given these examples, we believe that the adoption of flow techniques has a great potential to facilitate the synthesis of new scents with exciting odor profiles, while simplifying upscaling of the reaction to an industrial process.
